# Diel periodicity and visual cues guide oviposition behavior in *Phlebotomus papatasi*, vector of old-world cutaneous leishmaniasis

**DOI:** 10.1371/journal.pntd.0007165

**Published:** 2019-03-05

**Authors:** Tatsiana Shymanovich, Lindsey Faw, Nima Hajhashemi, Jimmie Teague, Coby Schal, Loganathan Ponnusamy, Charles S. Apperson, Eduardo Hatano, Gideon Wasserberg

**Affiliations:** 1 Department of Biology, University of North Carolina at Greensboro, 235 Eberhart Bldg., Greensboro, North Carolina, United States of America; 2 Department of Entomology and Plant Pathology, North Carolina State University, Raleigh, North Carolina, United States of America; National Institutes of Health, UNITED STATES

## Abstract

**Background:**

Phlebotomine sand flies are vectors of human leishmaniases, important neglected tropical diseases. In this study, we investigated diel patterns of oviposition behavior, effects of visual cues on oviposition-site selection, and whether these affect the attraction of gravid *Phlebotomus papatasi* (Scopoli), the vector of old-world cutaneous leishmaniasis, to olfactory cues from oviposition sites.

**Methodology/principal findings:**

To evaluate these questions, we conducted a series of experiments using attraction and oviposition assays within free-flight test chambers containing gravid females entrained under a 14:10 hrs light:dark photoperiod. By replacing sticky-screens or moist filter papers every three hours, we showed that oviposition site search occurs mainly in the latest part of the night whereas peak oviposition occurs during the early part of the night. Behavioral responses to olfactory oviposition cues are regulated by time-of-day and can be disrupted by transient exposure to a constant darkness photoperiod. Gravid females, but not any other stage, age, or sex, were attracted to dark, round oviposition jars, possibly resembling rodent burrow openings. This visual attraction disappeared in the absence of an illumination source. Egg deposition rate was not affected by jar color. Olfactory cues had the strongest effect when the visual cues were minimal.

**Conclusion and significance:**

Our study showed, for the first time, that visual cues in the form of oviposition-site color, lighting level, and photoperiod are important in guiding the oviposition behavior of phlebotomine sand flies. Furthermore, such visual cues could modify the flies’ sensitivity to olfactory oviposition cues. Our results suggest that chemosensory and visual cues are complementary, with visual cues used to orient gravid females towards oviposition sites, possibly at long- to medium-ranges during crepuscular periods, while olfactory cues are used to approach the burrow in darkness and assess its suitability at close-range. Implications to sand fly control are discussed.

## Author summary

Sand flies are vectors of human leishmaniases, an important neglected tropical disease. An alternative approach to the conventional delivery of an insecticide to the vector is to bring the vector to the insecticide using oviposition (egg-laying)-site attractants. Olfactory cues originating from organic matter have been identified as important sources of oviposition attractants. However, nothing is known regarding visual assessment of oviposition sites by sand flies. Also, little is understood about diel egg-laying patterns of sand flies. Finally, it is not known if visual cues and time-of-day may affect their sensitivity to olfactory cues. In this study, we investigated these questions in a series of lab experiments using free-flight cage arenas, with *Phlebotomus papatasi* (vector of cutaneous leishmaniasis). We showed that peak oviposition-site search and sensitivity to olfactory cues occurs mainly in the latest part of the night whereas peak oviposition occurs during the early part of the night. We demonstrated that only gravid females, but not any other stage or sex, were attracted to dark, round oviposition jars resembling burrow openings. Finally, we showed that sensitivity to olfactory cues is reduced in the presence of strong visual cues and in the absence of natural diel photoperiod.

## Introduction

Phlebotomine sand flies (Diptera: Psychodidae) transmit protozoan parasites (*Leishmania* spp.), as well as pathogenic bacteria (*Bartonella bacilliformis*) and viruses [1–3]. Most significant are the human leishmaniases that, following malaria and dengue, are the most pervasive vector-borne diseases. Leishamaniases are found worldwide in warm tropical, semi-arid, and arid environments. The population at risk is estimated at approximately 350 million people with annual incidence estimated at 1–1.5 million cases of cutaneous leishmaniasis, 500,000 cases of visceral leishmaniasis, and an estimated Disability-Adjusted Life Year (DALY) of 2.3 million [4, 5]. Leishmaniasis is distributed in the poorest regions of the world, and considered a neglected disease in terms of under-reporting, under-funding of research, and inadequate health care [6–8]. With no vaccine to protect against the etiologic agent, reduction of exposure to sand fly bites using insecticides is the most effective prevention measure [6, 9, 10]. This strategy is seriously constrained, however, by the evolution of insecticide resistance, which renders residual sprays less effective [10, 11]. Also, given the limited knowledge of sand fly breeding sites [12, 13], source reduction using biolarvicides has not been an effective approach [10, 14]. Hence, a more focused, targeted, and efficient control method is urgently needed [15].

An alternative approach to delivery of the insecticide to the vector is to bring the vector to the insecticide using attractants [16–18]. Oviposition-site attractants can provide the basis for a novel control and surveillance approach targeting gravid females that are typically responsible for pathogen transmission and population amplification. Oviposition traps have been used for the control of mosquitoes [19–23], but no such tool yet exists for the control of sand flies. Unlike other biting Diptera, sand flies develop in terrestrial rather than aquatic microhabitats [1, 3]. Eggs are typically laid in soil rich in organic material on which the larvae feed and develop through four instars before pupation and adult emergence [1, 3, 24, 25]. For both new- and old-world sand fly species, olfactory cues originating from organic matter of various sources have been identified as important sources of oviposition attractants and stimulants [26–29], and some of these cues were shown to be produced by microbes [30, 31]. Conspecific eggs are also attractive to sand flies [32–34], with dodecanoic acid identified as an oviposition stimulant for *Lutzomyia longipalpis* (Lutz and Neiva) [35].

Insects orient to oviposition sites using multi-modal sensory systems. Chemosensory cues play a central role as oviposition attractants and as lures in oviposition traps, especially for nocturnal insects [18, 20, 21]. However, visual cues may also play an important role, especially in diurnal and crepuscular insects [19]. For example, *Aedes albopictus* (Skuse) mosquitoes prefer to lay eggs in black plastic cups rather than in white, blue, or orange cups, or cups with black-and-white contrasting patterns [36], and *Aedes triseriatus* (Say) and *Anopheles* spp. visually prefer to oviposit in dark water [37, 38]. Although sand flies were shown to respond to visual cues [39–44], nothing is known regarding their visual assessment of oviposition sites. Many sand fly species, including our study species *Phlebotomus papatasi* (Scopoli), are tightly associated with animal burrows within which they find shelter, blood-feed, lay eggs and where coprophagic larvae feed on decaying fecal matter [13, 45]. Therefore, it is reasonable to hypothesize that gravid females would be visually attracted to dark round objects resembling burrow openings.

The timing of trap deployment may also affect the efficacy of oviposition traps because different species prefer to lay eggs at different times. The circadian oviposition behavior of mosquitoes has been relatively well studied, but not so for sand flies. For example, *Ae*. *aegypti*, a day-time biter, consistently laid eggs in the afternoon irrespective of ambient temperatures, and when maintained on a 12:12 hrs photoperiod it laid eggs just before light-out [46]. Given the nocturnal or crepuscular nature of sand flies [3, 47, 48] it is often assumed that they oviposit at night [49, 50]. Indeed, gravid females of *Ph*. *papatasi*, which typically breed in rodent burrows, were observed to exit the burrows in the early part of the night, suggesting that oviposition-site search activity may start early in the night [51]. In contrast, the majority of *Phlebotomus orientalis* (Parrot) gravid females were caught after midnight, suggesting that oviposition-related activity occurs mostly in the later part of the night [52]. However, both studies relied on indirect evidence based on locomotor activity, and to date nothing is known about the timing of oviposition-related activities in sand flies.

An important observation with mosquitoes was their integration of sensory inputs from olfactory cues and visual cues [53–55], and that it may vary during the diel cycle [56–59]. With sand flies, evidence for such interactions is scant. By studying circadian clock gene expression of *Lu*. *longipalpis*, researchers observed an evening peak that anticipated the lights-off cue [60]. With *Ph*. *papatasi*, adding a humidity source to a CDC trap increased capture only for white or clear traps but not for black traps [40]. To our knowledge, no studies with either mosquitoes or sand flies have documented such interactions in the context of oviposition behavior.

In this work, we used *Ph*. *papatasi*, a vector of *Leishmania major*, the etiological agent of zoonotic cutaneous leishmaniasis (ZCL) in the Middle East [3, 61]. We investigated its diel pattern of attraction to oviposition sites and timing of egg deposition, the effects of visual cues on oviposition-site selection and evaluated whether visual cues and photoperiod affect the attraction of gravid females to known olfactory oviposition cues. Specifically, our study focused on the following four aims: **(1)** Evaluate if time-of-day affects: (A) the timing of oviposition-site search activity, (B) timing of egg deposition, and (C) attraction to known olfactory cues; **(2)** Evaluate if transient changes in the photoperiod affect the attraction of sand flies to known olfactory oviposition cues; **(3)** Evaluate if visual cues, in the form of oviposition site color and illumination level, affect oviposition-site selection; and **(4)** Evaluate if visual cues affect the attraction of sand flies to known olfactory oviposition cues.

## Methods

### Ethics statement

As part of the sand fly colony maintenance, sand fly blood-feeding on ICR white mice was conducted at the SoBran research facility, Gateway University Research Park in North Carolina under SoBran IACUC Protocol number UNC-002-2016 dated 12/12/2016. This protocol was approved by the SoBran Gateway Animal Care and Use Committee; Chaired by Stephanie Strahan. This institution is USDA-registered and PHS-assured, therefore, SoBran’s animal care and use standards adhere to the Animal Welfare Act (USC 7, Sections 2131–2159, 1966, as amended) and Animal Welfare Regulations (9 CFR, Chapter 1, Parts 1–4); the PHS Policy on Humane Care and Use of Laboratory Animals USHHS, NIH, 2015); and The Guide for the Care and Use of Laboratory Animals (NRC, 8th edition), among others.

### General

#### (a) Insects and colony maintenance

*Phlebotomus papatasi* sand flies originating from Abkük, Turkey (April, 2004) were maintained following the mass-rearing methods described by Lawyer [49] and flies were blood-fed at SoBran (Greensboro, NC) on live anesthetized ICR mice (Envigo) (SoBran protocol # UNC-002-2016). Sand flies were maintained in incubators (Model: 6030–1, Caron, Marietta, Ohio) at 27°C, 85% RH. For aims 1, 2 and 4, flies were reared under a 14:10 hrs light:dark reverse photoperiod, with one hr of crepuscular light conditions (using a lamp containing 11W incandescent light bulb connected to an automatic timer) at the start of the light phase representing twilight at sunrise (dawn) and one hr at the end of the light phase representing twilight at sunset (dusk). For aim 3, flies were reared under 12:12 L:D reverse photoperiod. Given that our sand fly rearing protocol emulates closely natural photoperiod and larval rearing conditions, we expect the behavioral patterns exhibited by lab-reared flies used in this study to be representative of natural populations.

#### (b) Oviposition and attraction assays

**Fig 1:**
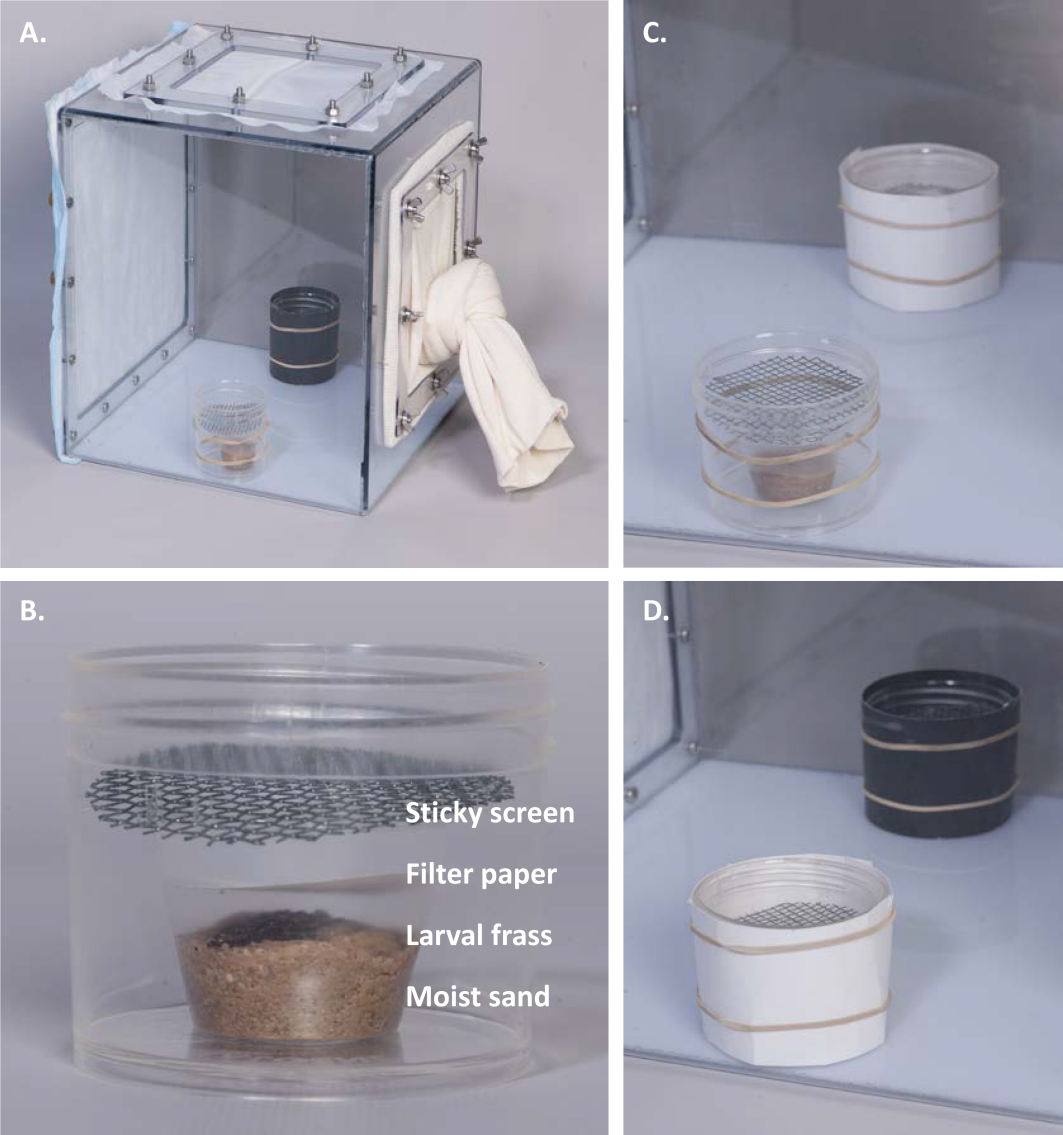
Design of test chambers and oviposition attractants used in this study. In paired-choice bioassays we placed a pair of small (125 ml), round, transparent jars in opposite sides of the cage (A). When testing for attraction of flies to visual or olfactory cues, we used sticky screen jars comprising a sand cup (a 30 ml disposable plastic cup containing 10 ml of wet autoclaved sand) with a sticky screen placed on top of it (B). When testing the effect of visual oviposition cues, we wrapped the treatment jar with either black or white poster papers and left the unwrapped jar as control (A, C). Bioassays evaluating the effect of sand fly sex or stage on their visual oviposition response were conducted using black and white jars (D). When testing for olfactory oviposition cues, we placed 0.1 g of larval frass in treatment jars (B) with no frass in the control jars. White filter paper was placed loosely above the sand (B) to control for possible visual effects. Image Credit: Daniel J. Smith.

We used transparent polycarbonate cages (30 x 30 x 30 cm) (Precision Plastics, Beltsville, Maryland) as free-flight test chambers (Fig 1A). For most experiments, we used mature (8–11 days-old) gravid (4–5 days post blood-meal) females that were acclimated overnight. To measure attraction or oviposition response, we placed a pair of oviposition jars in opposite sides of the cage (Fig 1A). An oviposition jar consisted of a 125-ml round, transparent jar (Nalgene, Rochester, New York) containing a 30-ml disposable plastic cup filled with 10 ml of wet autoclaved sand (hereafter, sand cup) (Fig 1B). When testing for attraction, we placed a sticky aluminum screen disk (6 cm diameter), made of an aluminum window-screen sprayed with adhesive (Tanglefoot, Model 91992-MI-001, Marysville, Ohio), on top of the sand cup. This sticky screen intercepted flies orienting toward the jar and prevented egg deposition (hereafter, sticky traps (Fig 1B)). By the end of the experiment, flies caught on each sticky screen were counted. In oviposition assays, sticky screens were not used; instead, in each sand cup, a moist filter paper (Model: 09-801-AA, ThermoFisher Scientific, Waltham, Massachusetts) was placed on top of the sand and flies were given three days to oviposit. Filter papers were then collected, and eggs counted. The bioassays were conducted in a walk-in environmental room maintained at 27 ^o^C and 64–67% RH. We placed Styrofoam dividers between adjacent cages to visually isolate the test cages.

#### (c) Measurement of light settings

To measure luminance, we used a hand-held Sky Quality Meter (SQM-L, Unihedron, Grimsby, Ontario, Canada) reporting luminance levels using magnitudes/square-arcseconds (mag/arcsec^2^) units, which were then converted to units of candela/meter^2^ (http://unihedron.com/projects/darksky/magconv.php). Measurements (*n* = 5) were taken at the center of the walk-in chamber at shoulder height and with the detector facing upward.

### Description of experiments

#### Experiment #1: Timing of oviposition-site search activity, egg deposition, and attraction to known olfactory cues

**Fig 2:**
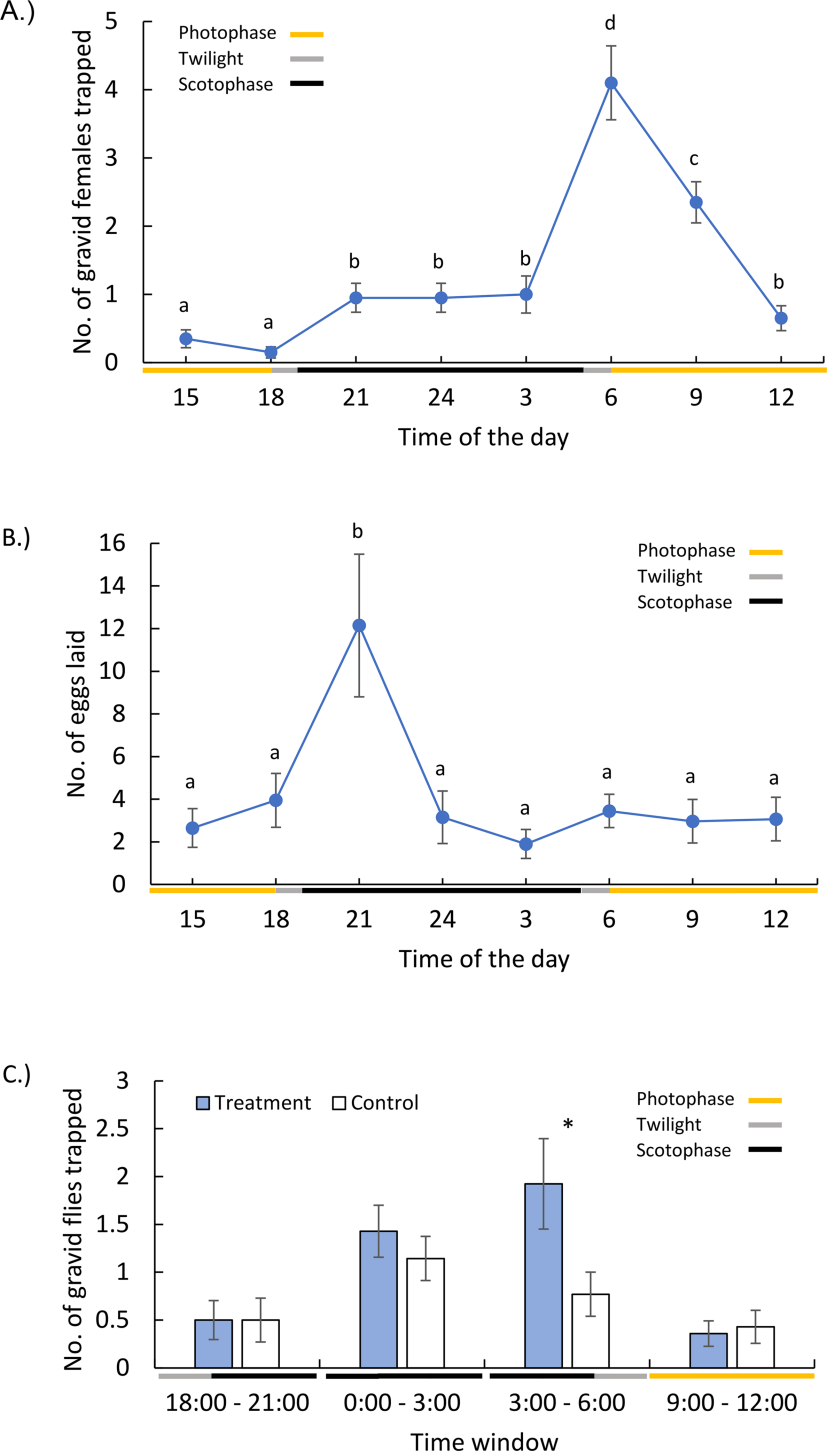
Diel periodicity of oviposition site search activity, egg deposition, and attraction to an olfactory cue of gravid sand flies. In oviposition site-search activity bioassays (A) and egg deposition bioassays (B), the time axis depicts time of the day (24-hours clock) with color bars indicating simulated lighting conditions during that time. (Error bars = SE). The olfactory cue in treatment jars (C) consisted of larval frass while control jars contained wet sand. Color bars indicate lighting conditions used during each time window (24-hours clock). (Error bars = SE). Asterisks indicate a significant Student’s paired *t*-test difference between the treatment and control jars within experimental cages (C) and different letters indicate significant differences among time periods (A, B). * *P* < 0.05.

Photoperiod settings within the walk-in environmental room used for these experiments corresponded to those used for sand fly rearing as described above. For timing of oviposition-site search activity (aim 1A), we used our standard holding cages (*n* = 10) containing 50 gravid females and a single, centrally placed, sticky screen jar with a sand cup containing 0.1 g of larval frass, a known oviposition attractive substrate [26] (Fig 1B). Every three hours, over a 48-hour period, the sticky screen in each cage was collected, replaced with a new screen, and flies caught on the screen were counted. For the oviposition timing experiment (aim 1B), we used the same holding cages (*n* = 10), each containing 50 gravid females. Each cage contained a single experimental jar, filled with 40 ml of water-saturated attractive mixture (equal ratio of larval frass, fresh larval food, and sand) on which a pair of round filter papers (60 mm diameter) was placed. The top filter paper was replaced every three hrs with a new moist paper and the eggs laid on it were counted over 90 hrs. To evaluate the diel periodicity of olfactory oviposition attraction (aim 1C), we used a paired-choice design with a pair of transparent sticky traps, with the treatment sand cup containing 0.1 g of larval frass and the control cup containing just moist sand, placed inside a standard holding cage containing 20 gravid females (*n* = 7). The timing of these 3-hrs-long oviposition-attraction bioassays was aimed to evaluate if the timing of sand flies olfactory attraction to a known oviposition attractant corresponds to the time of peak egg-deposition (early scotophase: 6–9 PM) or to peak oviposition-site search activity (late-scotophase: 3–6 AM) observed previously (Fig 2A and 2B). We included two additional reference time points: mid-scotophase (0–3 AM) and mid-early photophase (9–12 AM). This bioassay lasted 48 hrs with seven cages used for each time-window (repeated twice over the duration of the experiment). Luminance in the photophase, scotophase, and simulated twilight was measured at 6.54 ± 0.08 mag/arcsec^2^ (Mean ± SD; 261.5 candela/m²), 17.03 ± 0.05 mag/arcsec^2^ (0.016 candela/m²), and 13.40 ± 0.10 mag/arcsec^2^ (0.471 candela/m²), respectively. Thus, photophase was 16,344 times brighter than scotophase and 555 times brighter than twilight.

#### Experiment 2: Effect of photoperiod on attraction of sand flies to olfactory oviposition cues

In this experiment (aim 2), we evaluated if disruption of the natural photoperiod affected oviposition site selection; specifically, attraction to known attractive substrates for oviposition. Flies were entrained to a natural photoperiod (14:10 hrs light:dark, including 1 crepuscular hr at the start and end of the photophase) and tested either under these conditions or switched to constant darkness (0:24 hrs) for the 24 hr assay. We used two walk-in environmental chambers (27 ^o^C, 65–67% RH in both), one with a diel photoperiod and the other under constant darkness (16.43 ± 0.25 mag/arcsec^2^ [0.0289 candela/m²]). Each room contained holding cages (*n* = 15, 20 gravid females per cage) with a pair of transparent sticky-traps, one (treatment) with a known oviposition attractant (0.1 g of larval frass) and one without (control). The experiment ran for 6 hrs, between midnight and 6 am, which (as shown in experiment 1) represents the time-period when gravid females are most responsive to olfactory oviposition cues. At the end of this time-window, the flies trapped on the control and treatment jars were counted. Preference to the treatment jar was calculated as the proportion of flies caught in the baited jar compared with total flies trapped in the two jars combined. Response rate was measured as the total number of flies trapped at the treatment and control jars divided by the total number of flies per assay. The experiment was then repeated with the photoperiod-regime switched between the two environmental rooms.

#### Experiment 3: Effect of visual cues on oviposition-site selection

In this experiment, we evaluated the effects of oviposition jar color, illumination level, and adult sand fly stage and sex on sand fly attraction and oviposition responses. Pairs of transparent sticky traps placed at opposite sides of the cage were wrapped with either black or white poster paper or left unwrapped as controls (Fig 1). We tested the preference of gravid females for black versus transparent jars (Fig 1A), white versus transparent jars (Fig 1C), and black versus white jars (Fig 1D). The effect of light level on visual preference also was tested. In all three sets of experiments, visual attraction was evaluated under low light (not light-sealed room with two red incandescent bulbs, 17.77 ± 0.04 mag/arcsec^2^ [0.0084 candela/m²], *n* = 14), and dim light (using two 4W white incandescent night lights in addition to the low-light setting, 17.03 mag/arcsec^2^ [0.0166 candela/m²], *n* = 14). In the black versus clear jar experiment, we also included a trial conducted under complete dark conditions (light-sealed room with no internal light source, 22.99 ± 0.1 mag/arcsec^2^ [0.00007 candela/m²], *n* = 13) in order to test the hypothesis that visual attraction to black jars should disappear in the absence of a light source. To evaluate if the attraction of flies to black jars varied among different physiological stages and sexes we repeated the black versus white jar preference test (under dim-light) with young (1–2 days-old) non-blood-fed females (*n* = 14), mature non-blood-fed females (8–11 days old, *n* = 19), mature males (8–11 days old, *n* = 13), and young males (1–2 days-old, *n* = 13). We evaluated gravidity status of young, mature-blood-fed, and mature non-blood-fed females by dissecting ovaries of females of each group (≥ 20/group) and determining the fraction of females containing developed eggs in their ovaries. To evaluate the effect of visual cues on actual egg deposition, we used a pair of transparent jars filled with 40 ml of water-saturated sterile sand; either a white or black poster paper disk (38 mm diameter) was placed on top of the sand (*n* = 16). Gravid females (20 per cage) were allowed to lay eggs over 72 hrs on either the white or black poster paper disk. To confirm that the white and black poster papers did not provide differential olfactory cues, we performed an experiment using small pieces of the black and white papers (of equal mass as used for the experiments above) hidden inside 30-ml disposable cups wrapped laterally with aluminum foil and covered loosely with a filter paper (*n* = 21). Each cup was placed into a sticky screen jar containing 10 ml autoclaved wet sand. In addition, paper samples of each color were analyzed for amounts and composition of volatiles using gas chromatography (for detailed methodology see supplementary materials).

#### Experiment 4: Effect of jar color and illumination on the response of sand flies to olfactory cues

A pair of sticky traps of the same color—black, white, or clear—was placed into each holding cage. The treatment jar contained a sand cup with 0.1 g of larval frass (as olfactory cue) and the control jar contained a sand cup with no frass. Both sand cups were covered loosely with a filter paper to eliminate visual effect of the rearing medium (Fig 1B). A sticky-screen disk was placed in each jar on top of the sand cup. We used 20 gravid females per cage, and females were acclimated to the cage overnight. The sand flies caught on each screen were counted after 24 hrs. This experiment was repeated under three different light levels: dark, low-light, and dim-light as described above. At each experimental session, we used nine replicate cages for each jar color. Two replicate sessions were conducted for each light level.

### Data reduction and statistical analysis

In experiment #1, we evaluated the change in the number of flies caught on a sticky screen trap or the number of eggs laid on a filter paper over a diel cycle. We analyzed these longitudinal count data using a random-intercept negative-binomial regression model, with cage as the clustering factor [62].

We used paired Student’s *t*-test to compare the effect of treatment versus control within a cage. In order to compare the relative attraction of sand flies for the treatment, among treatment levels (e.g., illumination level, time window, jar color) we calculated the proportion of flies caught in the treatment jar compared with total flies trapped in the two jars combined (hereafter, preference). Values above 0.5 indicated preference for the treatment and values below 0.5 indicated aversion to it. Given that “preference” is a proportion, we analyzed these data using weighted logistic regression, with number of flies making a behavioral choice used as the weighting factor [63]. Analyses were conducted using Stata (StataCorp., College Station, TX).

## Results

### Experiment 1: Diel pattern of attraction to oviposition sites and egg deposition

#### (i) Timing of attraction to oviposition sites and oviposition

In no-choice assays, significantly more (*P* < 0.05) gravid sand flies were attracted to the oviposition site during the last three hrs of the scotophase and first three hrs of the photophase than at other times (Fig 2A). In contrast, oviposition activity peaked during the first three hrs of the scotophase, declined rapidly in mid-scotophase and remained low for the rest of the exposure period (Fig 2B). The association between peak egg deposition and peak attraction times with dusk and dawn, respectively, suggested that visual cues and circadian rhythms might mediate these behaviors.

#### (ii) Temporal changes of preference for olfactory cues

With two-choice assays (sand and larval frass vs. sand only), we assessed diel changes in preference of gravid sand flies for olfactory oviposition cues. During early scotophase no difference between the treatment and the control jars was observed (Fig 2C). Also, only 5% of flies made a behavioral choice, indicating low activity level. During mid-scotophase, activity level increased significantly (*t* = 3.21, *df* = 26, *P* = 0.002) to 12.8% and a non-significant trend (*P* = 0.412) towards preference for the frass-baited jar started appearing (Fig 2C). In the late scotophase, activity level slightly increased to 13.5% and preference for the frass-baited jar peaked and became statistically significant (Fig 2C). In the photophase (9:00–12:00), sand fly activity level dropped sharply (*t* = 3.33, *df* = 25, *P* = 0.0027) to 3.9% and preference to the frass-baited jar disappeared and even slightly reversed (Fig 2C).

### Experiment 2: The effect of photoperiod regime on sand fly attraction to a known olfactory cue

In this experiment, sand flies that were reared under a 14:10 light:dark photoperiod were tested for their olfactory preference to a known oviposition attractant (larval frass) under different photoperiod conditions: the 14:10 photoperiod to which they were entrained or constant darkness. Flies exposed to the entrainment photoperiod exhibited stronger attraction to the rearing medium compared with flies switched transiently to constant dark conditions with an average preference rate of 60.1 ± 3% and 49.0 ± 4%, respectively (*t* = 2.15, *df* = 59, *P* = 0.03) (Table 1). On average, under the standard 14:10 photoperiod, significantly more flies were trapped in the frass-baited jar than in the sand only control (4.52 ± 0.3 versus 3.06 ± 0.3 flies, respectively; paired-*t* = 3.32, *df* = 30, *P* = 0.001), whereas in assays conducted under constant dark conditions (0:24 light:dark) no significant difference was found between the two jar types (4.1 ± 0.5 versus 3.7 ± 0.3, respectively; paired-*t* = 0.612, *df* = 29, *P* = 0.27). This pattern was consistent in the two rooms in which the assays were conducted (Table 1). Also, no significant difference was found with respect to the response rates, with an average response rate of 45.3 ± 3% and 46.3 ± 3% under these respective conditions (*t* = 0.238, *df* = 59, *P* = 0.81). Here too, results were consistent in the two rooms in which the experiment was conducted (Table 1).

**Table 1:** Effect of photoperiod regime (diel entrainment cycle or constant darkness) on the olfactory preference and response rate of gravid females to oviposition jars containing larval frass compared to control jars.

Round	Room	Assay photoperiod (L:D)	Preference for frass (%)	Response rate (%)
1	1	14:10	61.5 ± 4 ^A,^**	48.3 ± 3^A^
1	2	0:24	54.2 ± 7 ^A,ns^	49.3 ± 5^A^
2	1	0:24	43.8 ± 4 ^a,ns^	43.2 ± 3^a^
2	2	14:10	61.8 ± 5 ^b,^*	43.4 ± 4^a^

Footnote: Preference is depicted as the mean (± SE) percentage of flies trapped in the treatment jar compared with total number of flies trapped in both jars. Preference above 50% indicates attraction and below 50% indicates repellence. Response rate was measured as the total number of flies trapped at the treatment and control jars divided by the total number of flies per assay (X100). Letters are used to compare the preference for larval frass between the two photoperiod regimes, with upper-case letter for round 1 and lower-case letters for round 2. Different letters indicate significant difference in preference between the two photoperiod regimes. Asterisks denote differences between the treatment and control jars for the paired-design bioassays: ** *P* < 0.01, * *P* < 0.05, ns *P* > 0.05

### Experiment 3: Effects of visual cues on oviposition-site selection

#### (i) Black and white papers do not emit differential olfactory cues

**Fig 3:**
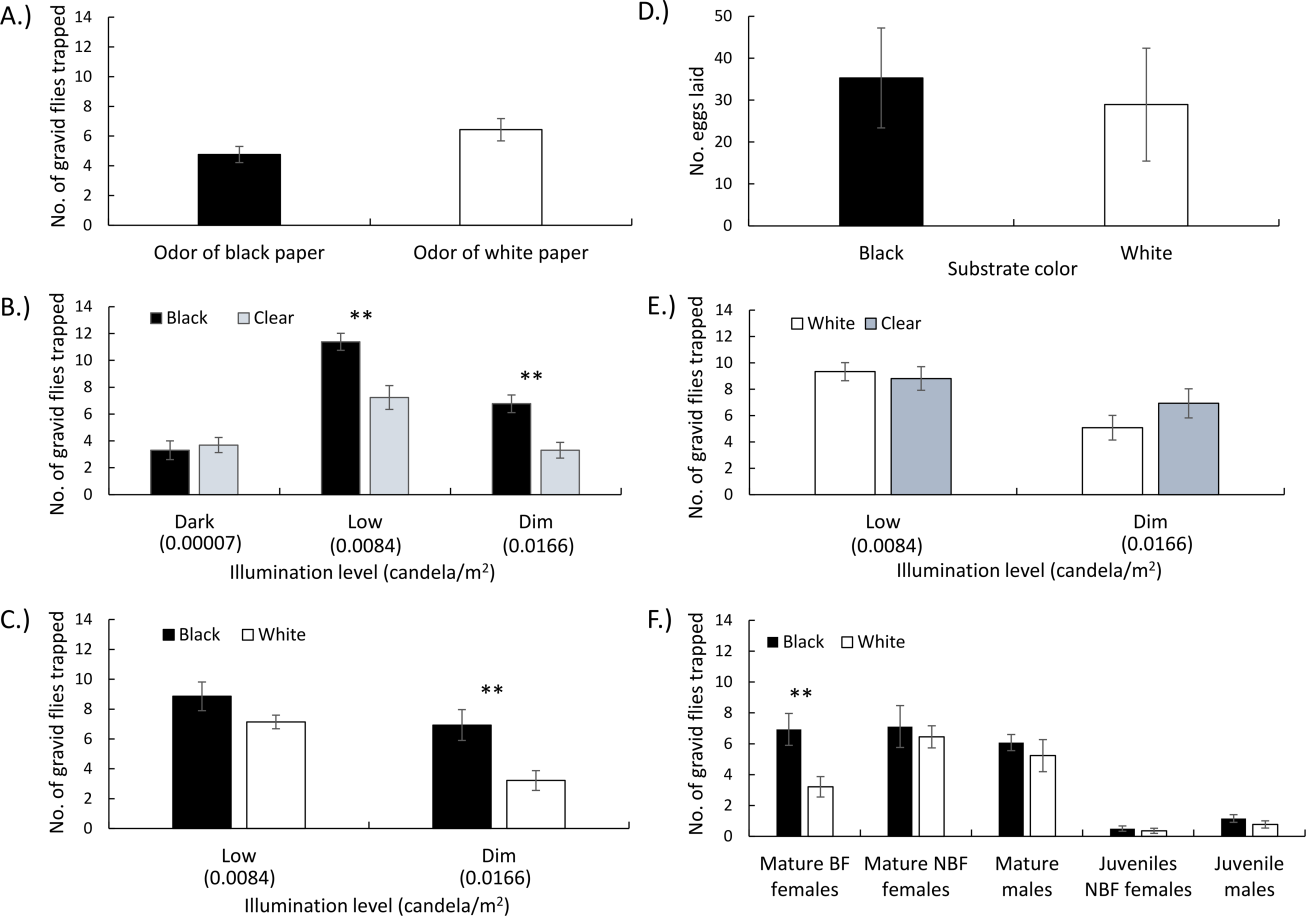
Effects of visual cues on sand fly oviposition site-selection behavior. (A) Effect of paper odor on gravid sand fly oviposition attraction. (B) Evaluation of gravid sand fly visual attraction to black jars versus clear jars under three illumination levels. (C) Evaluation of gravid sand fly visual attraction to black jars versus white jars under two illumination levels. (D) Comparison of sand fly egg deposition rate between black and white papers. (E) Evaluation of gravid sand fly visual attraction to white jars versus clear jars under two illumination levels. (F) Effect of age and sex on the attraction of sand flies to black versus white jars. (BF = blood-fed, NBF = non-blood-fed). Error bars = SE. Asterisks indicate a significant paired-*t*-test difference between treatment and control within experimental cages * *P* < 0.05, ** *P* < 0.01.

We found minor qualitative differences in the volatile compounds emitted from black and white papers (S1 Fig). In addition, by calculating the total area of analyte peaks in chromatograms, we determined that the amount of volatiles emitted by black papers was 18% higher than white papers. In order to confirm that the response of gravid females was not affected by these minor differences in volatile composition, we tested their preference to odors emitted from black and white papers while eliminating visual cues. We found no significant difference between the number of flies caught (paired-*t* = -1.63, *df* = 21, *P* = 0.11) with a slight non-significant attraction to the white paper odors (Fig 3A). This result suggested that paper-derived chemosensory cues were not involved in attraction to jars.

#### (ii) Response to jar color and light level

In two-choice attraction assays, gravid sand flies exhibited significant preferences for black oviposition jars over clear jars under low and dim luminance levels but not in complete darkness (Fig 3B). These results indicated that sand flies preferred dark oviposition sites and suggested that they visually differentiated colors, hues or contrast. In complete darkness, however, sand flies could not use visual cues to orient to oviposition sites and thus exhibited no preference for black or clear jars. Similarly, gravid sand flies preferred black jars over white jars, but only in dim light from a white incandescent bulb and not in low light from a red incandescent bulb (Fig 3C). In oviposition assays, however, there was no significant difference (paired-*t* = 0.387, *df* = 31, *P* = 0.70) between the number of eggs oviposited on a black paper disk and on a white paper disk (Fig 3D), suggesting that attraction and orientation preferences were visually guided, but not oviposition. In contrast with black jars, gravid sand flies did not differentiate white jars from clear jars under either dim (paired-*t* = 1.498, *df* = 13, *P* = 0.16) or low light (paired-*t* = 0.418, *df* = 20, *P* = 0.68) conditions (Fig 3E).

#### (iii) Effect of stage and sex on visual orientation

The preference for black jars was clearly stage-specific. Only mature blood-fed gravid females exhibited significant preference for black over white jars (Fig 3F). In contrast, mature non-blood-fed females did not exhibit a significant preference for black jar (paired-*t* = 0.47, *df* = 17, *P* = 0.64) (Fig 3F) even though 74% of these were found to be autogenically gravid (Fig 3F). Mature blood-fed females were 100% gravid and produced an average of 46.9 eggs (±3.48) compared with 16.37 (±1.08) mature non-blood-fed females (*t* = 6.41, *P* < 0.0001). None of the young non blood-fed females were gravid. Mature males, also did not exhibit significant attraction to black jars (Fig 3F). Similarly, neither young unfed females nor young males exhibited significant preference towards black jars (Fig 3F). Note, however, that all these non-blood-fed females or males exhibited a slight, non-significant, attraction trend towards the black jars (Fig 3). Notably, mature blood-fed (36.3 ± 5%) (*t* = 6.32, *df* = 26, *P* < 0.0001) or unfed females (45.6 ± 6%) (*t* = 5.77, *df* = 31, *P* < 0.0001) had a significantly higher response rate compared with young unfed females (2.8 ± 0.9%). Similarly, response rate of mature males (60.0 ± 4.0%) was significantly higher compared with that of young males (9.7 ± 1.6%) (*t* = 10.18, *df* = 13, *P* < 0.0001).

### Experiment 4: Interaction of visual cues, olfactory cues and illumination

**Fig 4:**
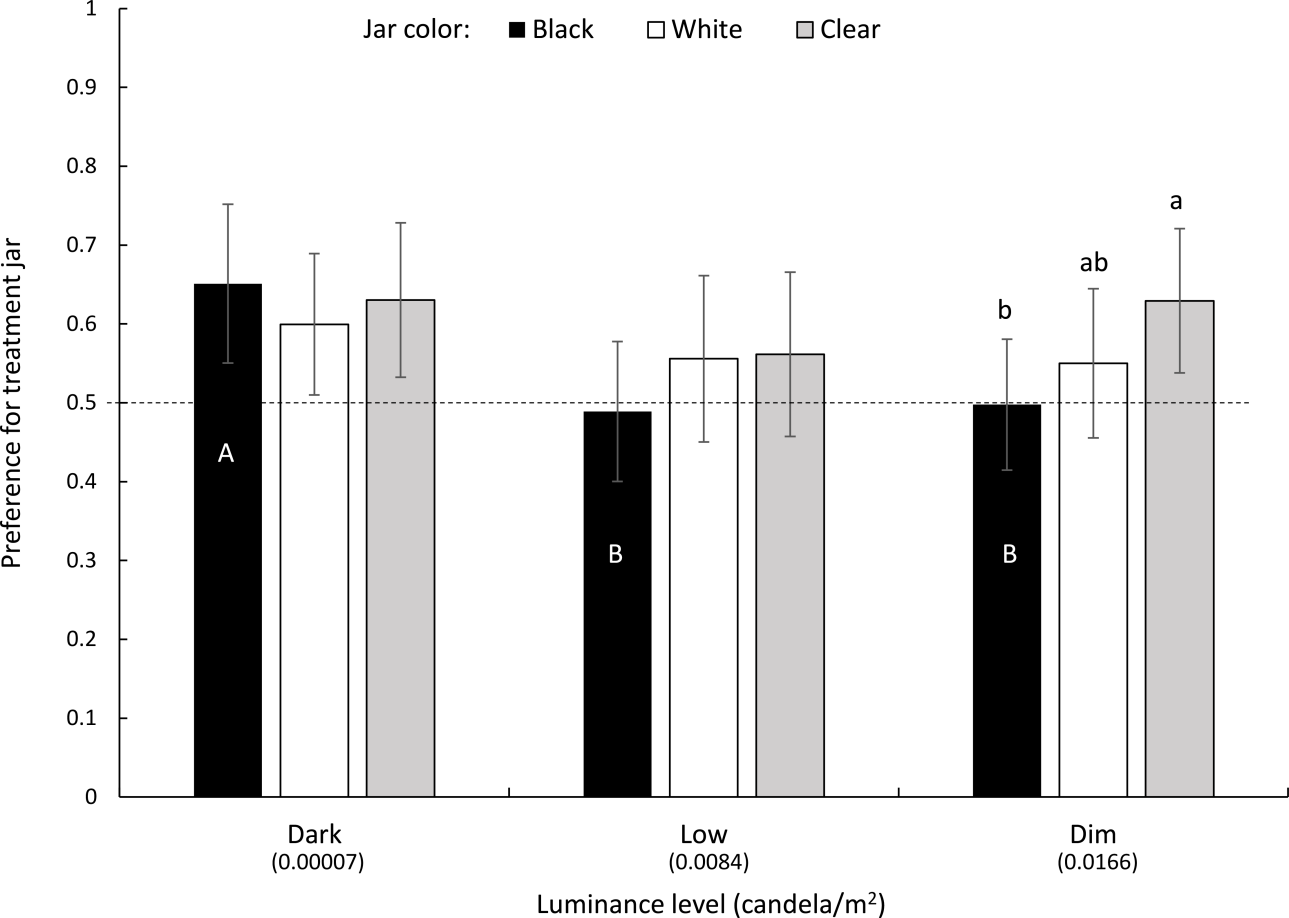
Effect of illumination level and jar color on the preference of gravid sand flies to jars containing larval frass medium. Jar color included black, white, and clear. Preference was measured as the fraction of flies trapped in the treatment jar of the total flies trapped in both jars combined. Preference values not significantly different from 0.5 (marked as a dashed line) indicate no preference, while values significantly larger indicate preference for the baited jar. Error bars = 95% confidence intervals. Upper-case and lower-case letters indicate difference among illumination levels and jar colors, respectively.

We conducted two-choice assays, using free-flight cages containing a pair of sticky traps of the same color with one jar containing larval frass (olfactory cues) and the other jar serving as control (moist sand only). Under complete darkness, gravid females exhibited clear olfactory attraction to the treatment jar irrespective of jar color (Fig 4). As light level increased to low-level (a 120-fold increase in brightness), olfactory attraction to the frass-containing jars decreased and became non-significant for all jar colors (Fig 4). This decrease was particularly steep and statistically significant for the black jars where attraction disappeared altogether (Fig 4). For other jar colors, preference showed a non-significant decreasing trend. No significant difference was found among jar colors (deviance = 2.89, *df* = 2, *P* = 0.23). With further increase in luminance (dim lighting level, 2-fold brighter than low-light), olfactory attraction to frass-baited jars remained low and non-significant for both black and white jars. For transparent jars, however, preference for the frass-baited jars increased and became significantly greater than for the black jars (paired-*t* = 2.73, *P* = 0.014) (Fig 4).

## Discussion

In this study, we investigated the diel pattern of attraction of *Ph*. *papatasi* to oviposition sites and egg deposition, effects of visual cues on sand fly oviposition-site selection, and whether these two variables affect response of gravid sand flies to known olfactory oviposition cues.

### (a) Gravid sand flies search for suitable oviposition sites during the late part of the night but oviposit mainly in the earlier part of the night

Essentially, nothing was known about the timing of oviposition-related behaviors in sand flies. With sand flies being predominantly nocturnal or crepuscular [3, 47, 48], it was assumed that they oviposit during the dark phase [49, 50]. However, the questions as to when they search for oviposition sites and when they actually deposit their eggs have remained unanswered. With specially-designed exit-entrance traps, Yuval and Schlein (1986) observed that gravid *Ph*. *papatasi* females constituted a large fraction of flies exiting rodent burrows in the early part of the night, suggesting that oviposition-site search activity is more likely to occur early in the night [51]. In contrast, our results show a clear peak in activity related to oviposition-site search activity in the last three hrs of the scotophase that seems to also spill-over into the dawn and early hours of the morning. Furthermore, our results indicate that this time-period (the last three hours of the scotophase) is also when olfactory attraction to known oviposition attractants peaks. Obviously, our experiments should be repeated using field-caught or freshly produced lab-reared flies under field conditions. However, assuming our findings extend to natural sand fly populations, these results suggest circadian regulation of oviposition site-search behavior that affects the sand fly’s activity level and possibly sensory responsiveness to olfactory and visual oviposition cues. Why oviposition site-search activity is delayed until the later part of the night is yet to be determined. It is possible that, initially, gravid females need to search for a sugar-meal in order to replenish their energy reserves before switching to an oviposition site-search mode. This speculation is consistent with an observation by Yuval and Schlein (1986) showing that a high proportion of females entering burrows at the later part of the night were sugar fed, compared with those exiting burrows in the earlier part of the night [51].

With respect to timing of egg-laying, we observed a distinct peak of oviposition during the first three hours of the scotophase (which also included 1 hr of dusk) and lower oviposition rates before or after. These results highlight the adaptive value of circadian organization, which enables the coordination of metabolic processes and behaviors, including the expression of sensory receptors, relative to local entrainment conditions. The circadian organization of *Ph*. *papatasi* gravid females may be as follows: gravid females initially search for a sugar-meal during the night, then they switch to actively seeking a suitable oviposition site (often a rodent burrow) late in the night, they shelter within the burrow during the day, and then, possibly triggered by declining lights, other environmental changes such as relative humidity, or activity of the rodent host, they initiate egg-deposition at the beginning of the following night. Such a pattern is consistent with the behavior of many nocturnal mosquito species that tend to oviposit during dusk and early evening [19]. It is possible that a resting period within the burrow, before oviposition, enables females to complete egg maturation, mate, or even take supplementary blood meals from the resting host within the burrow. We cannot, however, discount the possibility that gravid females might also be laying eggs during the day within the dark burrow, and this possibility will be evaluated experimentally in the future.

### (b) Response to olfactory oviposition cues is affected by time of the day and photoperiod

Attraction to a known olfactory substrate (larval frass) was shown to vary along the diel cycle with few flies attracted during the photophase or early in the scotophase, a slight increase towards midnight, and many flies attracted to frass during the last three hours of the night. Furthermore, our results indicate that the level of olfactory attraction of gravid sand flies to an attractive substrate is affected by the photoperiod regime to which they are exposed. Specifically, sand flies entrained under a natural photoperiod (14:10 hrs light:dark, including 1 crepuscular hr at the start and end of the photophase) exhibited greater attraction to frass under these conditions than when switched to constant darkness (0:24 hrs) for the 6-hr assay. On the other hand, activity levels, as expressed in terms of the flies’ response rate, did not differ between the two photoperiod regimes (Table 1). These results suggest that visual cues associated with the dawn crepuscular period and early photophase may affect the flies’ oviposition site-selection behavior by modifying their sensory responses to volatile olfactory cues.

### (c) Gravid and blood-fed sand fly females, but not any other stage or sex, are visually attracted to black round objects possibly resembling openings of rodent burrows

We validated experimentally that preference for the black jars was due to visual cues and not due to olfactory attraction to potential volatile components of the black paper, because flies did not differentiate between jars containing concealed black or white papers. Gravid females clearly preferred black jars over transparent (clear) jars or white jars, but they failed to differentiate between white and clear jars. To further explore the role of visual cues, we performed the black-versus-clear-jar choice experiment at three illumination levels: complete darkness, low light (simulating dark moonless night conditions), and dim light (simulating moon-lit night conditions). In the absence of any light, jars of different colors should not be visually differentiable, and indeed, sand flies did not exhibit a preference for black or clear jars. In contrast, in low and dim light gravid females clearly preferred the black jar over the clear jar. Likewise, gravid females preferred the black jar over the white jar in dim light, but showed no preference between clear and white jars.

We also discovered that the effect of the visual cue pertains only to the oviposition site-search phase but not to the actual egg-laying phase. When provided with the choice of laying eggs on a wet black versus a wet white paper no difference in egg numbers was found. This observation is consistent with previous studies on both old- and new-world sand flies indicating that oviposition stimulation is triggered by chemosensory cues of fecal or conspecific origin [26, 27, 29–31].

Most striking was the fact that attraction to black jars over white jars was only exhibited by mature gravid blood-fed females. In contrast, non-blood-fed mature females, 74% of which were autogenous, did not exhibit such visual attraction (although a slight trend in that direction was suggested). This suggests that the level of female gravidity (% gravid) and fecundity (per-capita rate of egg production), which were shown to be higher in blood-fed females, are associated with the sensory acuteness of these females to visual oviposition cues. Visual attraction of adult males to black jars could be expected to mirror that of gravid females if mate-seeking males would await incoming gravid females in burrow entrances. The non-significant attraction trend we observed might be consistent with such a trend, but more study on that is obviously required. We also observed that activity level of young flies of both genders was considerably lower than that of mature flies, suggesting that activity of young flies may be stimulated by other cues such as those indicating presence of sugar- or blood-meal that are more relevant to flies at this stage [17, 51]. Overall, this stage-specificity of responses to visual cues suggests that the black jars are perceived by gravid females as a visual oviposition cue and not as a generic cue. This result somewhat differs from findings that host-seeking *Ph*. *papatasi* flies were attracted to CDC traps where the collection cup was wrapped in black paper [40]. Attraction of gravid flies to black round jars that might be perceived as rodent burrow openings is consistent with our knowledge of the biology of *Ph*. *papatasi* that in arid areas are highly dependent on rodent-host burrows (typically *Psammoys obesus* or other *Meriones* spp.) as suitable oviposition and shelter sites [64, 65]. The slight, non-significant affinity of sand flies of both genders and age/stages to black jars suggests a certain generality of this cue. Yet, the strong attraction of gravid females to a dark and round object suggests that they have evolved to become highly attuned to such a visual cue, which would facilitate the detection of sparsely distributed oviposition sites in arid environments.

### (d) Responses to olfactory oviposition cues are diminished in the presence of visual oviposition cues

In complete darkness, gravid females exhibited significant olfactory attraction to used larval frass, a known source of volatile oviposition attractants [26, 27], irrespective of oviposition jar color. As the light level increased, however, the degree of attraction to the frass-containing jar decreased or even disappeared altogether as visual cues became more apparent. This effect was particularly evident with black jars, which, as described above, provided the strongest visual cue to gravid females. Our results suggest that although visual and olfactory cues are integrated in the central nervous system to drive behavior, different sensory modalities take precedence during the orientation sequence. Such effects have been observed with mosquitoes [19]. In the confines of the relatively small, dimly lit, arena under our experimental conditions, visual cues appear to prevail over olfactory cues. Under natural conditions, however, we suspect that a cryptic dark burrow entrance on the surface of a relatively poorly lit ground might not be as apparent as a jar with a black vertical silhouette against a light-colored background, as used in our assays. We further suspect that chemosensory and visual cues are complementary, with visual cues used to orient gravid females towards potential oviposition sites at long-to-medium ranges during the crepuscular periods, while olfactory cues are used to approach the burrow in darkness and assess its suitability at close-range. Direct evaluation of the complementary effect of these cues would require a separate factorial-design experiment, including manipulation of the visual apparency (signal-to-noise) of the burrow entrance.

### (e) Implications for sand fly control and future experimentation

Results of this study add important novel insights for our understanding of the basic biology of Phlebotomine sand flies and have important implications for management of sand fly populations. First, in the context of the development of an oviposition trap, it is clearly necessary to take visual cues into account. Such a trap should be black and round resembling a rodent burrow entrance. Optimizing trap shape and design is an important next step, and of particular importance are its vertical aspect and apparency relative to the ground terrain. Then, visual cues should be complemented with chemosensory cues that include olfactory stimuli to guide the flies to the burrow and contact and gustatory cues to arrest females and stimulate oviposition. Our results show that visual cues might transcend olfactory cues, but this might not be the case under natural light and landscape conditions. Our findings that oviposition site search activity occurs in the late scotophase would suggest that traps should be activated in the field during the second part of the night and early morning. This would require that traps either be deployed in the later part of the night or that the olfactory lure is operational throughout the night. Nevertheless, these results require confirmation in the field with traps or rodent-burrow monitors under various natural-light conditions. Finally, results of this study contribute three important features and conditions for optimizing bioassays for further studies of sand fly oviposition site-selection. First, when screening olfactory attractants, visual cues should be minimized and probably transparent jars would serve this purpose best. Second, attraction bioassays with gravid sand flies should be implemented in the later part of the scotophase under minimal light, conditions under which chemosensory information takes precedence over visual cues. Finally, flies should be reared and maintained using a natural photoperiod representative of summer conditions to optimize the sensitivity of bioassays. These recommendations are already implemented in current studies in our lab.

## Supporting information

S1 Text GC-FID analysis of odors from black and white papers. (PDF)

S1 Data Data file of results of experiment 1. (XLSX)

S2 Data Data file of results of experiment 2. (XLSX)

S3 Data Data file of results of experiment 3. (XLSX)

S4 Data Data file of results of experiment 4. (XLSX)

S1 Fig Chromatograms of headspace collections from white and black papers, and empty borosilicate containers (blank). Traces are mean of GC recordings (n = 2).
(PDF)
