# Supplementary material for: Diel periodicity and visual cues guide oviposition behavior in Phlebotomus papatasi, vector of old-world cutaneous leishmaniasis
Source: PLoS Negl Trop Dis. 2019 Mar 5;13(3):e0007165. doi: 10.1371/journal.pntd.0007165 (PMC6420040; doi:10.1371/journal.pntd.0007165)
Supplement: S1 Text — (PDF) [file pntd.0007165.s001.pdf]

S1. Odors from Black and White Paper

Materials and Methods

Odor Collection and Analysis

Rectangular pieces of paper (3.5 x 2.0cm) were inserted into 4 ml borosilicate vials, capped and odors accumulated for 30 min at room temperature. Odors were collected with a solid phase microextraction (SPME) fiber coated with polydimethylsiloxane/carboxen/divinylbenzene (24 ga, Supelco, Bellefonte, PA, USA). A fiber was inserted through the autosampler cap, exposed to the headspace of the vial for additional 30 min, retracted and injected in a gas chromatography-flame ionization detector (GC-FID) system.

Paper odors were analyzed using an Agilent 7890A gas chromatograph with a DB-5 Agilent capillary column (30 m x 0.25 mm x 0.25 μm) and a FID for quantification. The SPME fiber was injected into a 250°C inlet operated in splitless mode. The split valve was turned on after 0.5 min. The oven temperature program was as follows: 40°C for two min, increased at 10°C/min to 250°C. Hydrogen was used as the carrier gas at constant flow (average linear velocity = 35 cm/sec) and the FID was set at 260°C.

Results

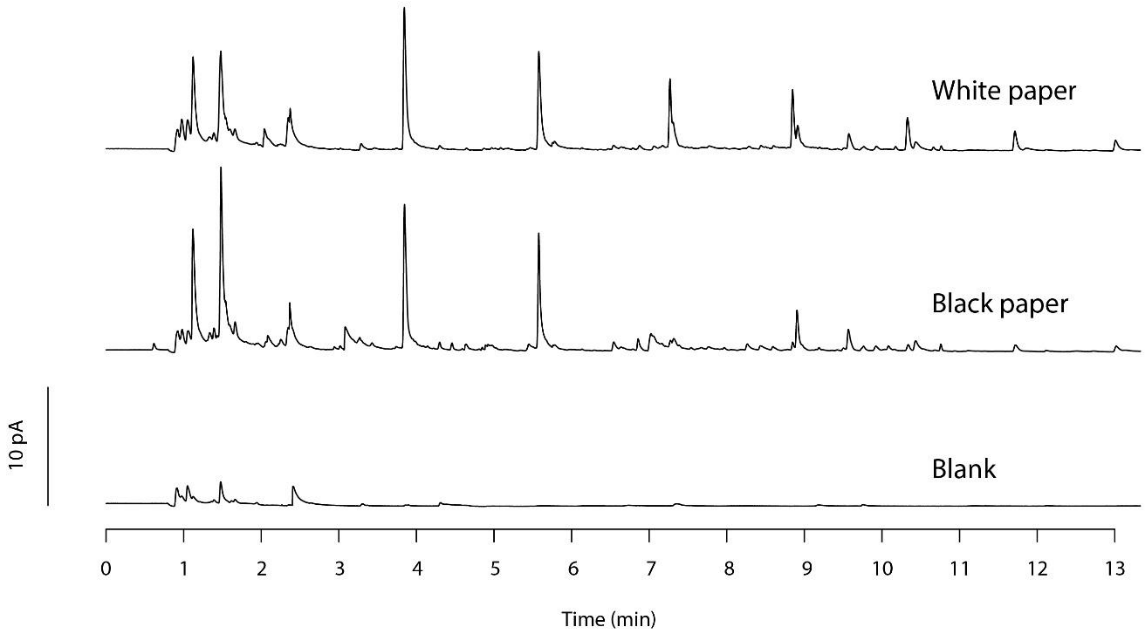

Figure A. Chromatograms of headspace collections from white and black papers, and empty borosilicate containers (blank). Traces are mean of GC recordings (n = 2).
